# Mapping the Dutch vaccination debate on Twitter: Identifying communities, narratives, and interactions

**DOI:** 10.1016/j.jvacx.2019.100019

**Published:** 2019-03-21

**Authors:** Roel O. Lutkenhaus, Jeroen Jansz, Martine P.A. Bouman

**Affiliations:** a Center for Media & Health, Gouda, the Netherlands; b Erasmus Research Centre for Media, Communication and Culture (ERMeCC), Erasmus University Rotterdam, the Netherlands

**Keywords:** Vaccine hesitancy, Health communication, Social media, Network analysis

## Abstract

In recent years, vaccination rates in the Netherlands have declined slightly, but steadily. The Dutch National Institute for Public Health and the Environment (RIVM) commissioned a Committee for Vaccine Willingness (VWC) to study the societal context of the decline. One of the societal contexts is the Internet, where audiences discuss vaccination and refer to sources of health-related information of varying quality.

Working for the VWC, we have explored the Dutch vaccination debate on Twitter in order to: (1) identify online communities in the vaccination debate, (2) identify vaccine-related narratives; and (3) understand how the online communities interact with each other. We identified seven different communities, including (public) health professionals, writers and journalists, anti-establishment, and international vaccination advocates.

The debate is spearheaded by the writers & journalists community, while the health- and anti-establishment communities try to influence it. The health community circulates facts, figures and scientific studies, while negative messages about vaccination – either from a homeopathy or conspiracy perspective – are most prevalent in the anti-establishment. The facts and figures shared by the health community hardly reach other communities, whereas the myths introduced by the anti-establishment do spill over to other communities. Our study provides further evidence that negative perceptions about vaccination might be rooted in a wider sentiment of distrust of traditional institutions.

We argue that Dutch health organizations should try to address questions, doubts, and worries among the general audience more actively, and present scientific information in a simpler and more attractive way.

## Highlights

• Analysis of the Dutch Twitter debate on vaccination using digital methods.
• Identification of online communities and mapping their perceptions and interactions.
• Communities include (but not limited to) health professionals and anti-establishment.
• Anti-establishment most negative about vaccination; health info hardly reaches them.
• Scripts to retrieve, process, and analyze Twitter data available for future research.

## 1 Introduction

In recent years, vaccination rates in the Netherlands have slightly but steadily declined [1]. Although the vaccination rates remain above critical levels, the Dutch National Institute for Public Health and the Environment (RIVM) commissioned an External Advisory Committee on Vaccination Willingness (VWC) to study the societal context of the decline, and to advise the RIVM on strategies to address it [2].

One of the societal contexts that the VWC set out to explore is the role of the Internet. Online platforms are leading audiences to sources of health-related content of varying quality [3] and social media have given rise to communities of vaccine advocates and anti-vaccine activists who use Web 2.0 services to circulate their message [3], [4], [5]. Several studies have associated *anti-vaxx* communities with the emergence of a postmodern paradigm in health care in which people favor their own interpretations over evidence-based facts and question the legitimacy of traditional institutions [4], [6], [7]. The VWC was especially interested in the role that these communities play in the vaccination debate in the Netherlands. Which communities are engaged in the vaccination debate and what role do they play in the Dutch media landscape? How do they interact with others, with the government, and with knowledge institutes such as RIVM?

Commissioned by the VWC, we investigated how Dutch Twitter users discuss vaccination [8]. The main research objectives were to: (1) identify the different communities and understand their backgrounds; (2) identify the most important vaccine-related narratives; and (3) examine how the communities interact by exposing the ways in which narratives flow through the network.

### 1.1 Theoretical background

Web 2.0 has enabled audiences to create and gather in public spaces beyond the realm of mainstream media [9]. There, media consumers use Web 2.0 services to relate to so-called social domains, which are figurations of organizations and individuals engaging with each other on a common topic or issue [10], [11] like vaccination. Sometimes, online communities use their voice for a specific reason, such as raising awareness about the alleged side-effects of vaccination. Multiple communities engaging on the same topic can have contrasting interests, and in those cases they can become allies or rivals and, in publicly negotiating their interests, their voices will reinforce or oppose each other [12].

It is important to study vaccine-related conversations across these communities, because discussions can instill norms and affect perceptions that may ultimately impact the vaccine-related decisions made by individuals [13]. Perceptions about vaccination may vary strongly across communities and two mechanisms have been associated with exaggerating those differences. First, online platforms tailor their content feeds to individual preferences, resulting in a *filter bubble* for individual end-users where they are selectively exposed to media content aligned with their interests and beliefs [14], [15], [16]. Members of anti-vaxx communities, for example, are automatically exposed to more negative representations of vaccination. Second, so-called *echo chambers*, or the process of one’s preexisting opinions constantly being reinforced by likeminded peers, ultimately contributes to polarization between communities of concurring audiences [17], [18].

The extent to which an individual is exposed to vaccine-related information, or is subjected to vaccine-related norms, depends on where he or she is situated in the network. As an online equivalent of a stakeholder analysis, it is therefore important to identify online communities and understand how they perceive vaccination and why.

## 2 Method

We have employed a mixed-method approach to identify different online communities in the Dutch vaccination debate, disentangle their voices, and inspect how information travels within and across communities. In this section, we will outline how we have retrieved, processed, and analyzed our data. The scripts to gather and process the data draw extensively from both Kearney’s *rtweet* package [19] and the *igraph* package [20] and have been made available publicly via GitHub^1^.

### 2.1 Retrieving tweets

We retrieved all Dutch Twitter messages (*statuses* or *tweets*) written between 07 and 28-17 and 12-02-17 that included the words: *‘vaccinatie’*, *‘vaccineer’*, *‘vaccineert’*, *‘vaccineren’*, *‘vaccineerde’*, *‘vaccineerden’*, *‘gevaccineerd’*, *‘gevaccineerden’*, *‘vaccin’*, *‘vaccins’*, *‘inenting’*, or *‘inenten’*^2^. This produced a collection of 2869 tweets by 1684 unique users.

Many of these tweets resulted from (multiple) interactions between users. For example, 823 of our 2,869 original tweets (28.7%) were replies, 414 (14.4%) were retweets, and 249 (8.7%) were quotes. Many of these statuses would not have been written without an original tweet to retweet, quote, or reply to. As we wanted our data to reflect this context, we retrieved the (chains of) tweets that triggered the retweets, quotes, and replies in our initial set, resulting in 2,437 extra tweets by 1,197 unique users, of whom 324 unique users were present in our initial data set. This led to a sample set of 5,306 unique messages written by 2,557 unique users.

### 2.2 Retrieving the network

Just a small section of all registered Twitter users actively tweet; many users merely lurk or are inactive [21], [22]. However, connections between non-tweeting and tweeting users make up a large part of the digital infrastructure that facilitates the circulation of vaccine-related content and can be used to reveal the underlying social context. Therefore, for each of the unique Twitter accounts in our earlier-retrieved set of tweets (the *authors*), we retrieved all their *followers* (accounts following the authors: 34,135,154) and *followees* (accounts followed by the authors: 1,288,618).

We were interested in identifying online communities based on shared interests (*who the authors are following*) and shared audiences (*who the authors are followed by*). We therefore excluded followers and followees who were not connected to at least 15 authors. We determined this cut-off point by examining the distribution of the number of connections with authors and arrived at our ultimate network size to stay within the limits of what our hardware and software were capable of handling in terms of visualization. Ultimately, our network included 121,623 Twitter accounts and 3,706,124 connections.

### 2.3 Analysis

To analyze our data, we iterated through a cycle of four steps, combining the merits of quantitative and qualitative analyses.

#### 2.3.1 Step 1: Community detection

We used the *Louvain algorithm* to detect communities in the network of authors and their wider social context. The Louvain algorithm is known as a fast, but relatively accurate, method to detect communities in large-scale networks [23]. We visualized the network using *Gephi* [24]. We only retained the communities that included at least 1% of all the network’s users.

#### 2.3.2 Step 2: Text-mining

We verified whether each community comprised groups of likeminded audiences by analyzing the profile texts of all users. We analyzed the profile texts of *authors* as well as the profile texts of the *followers* and *followees* they have in common. Consequentially, the analyzed profile texts do not reflect alignment in the vaccination debate, but alignment in the wider media ecology.

Next, we examined the vaccination-related tweets in the different communities to distinguish different vaccination-related narratives. The results reflect how the *authors* in each community engage on vaccination. The results also show to which narratives the members of each community were most likely exposed.

To analyze the tweets, we first filtered stop words and stemmed the text using the *MBSP* text-analysis system [25], which can process the Dutch language. Then, using the *tidytext* package [26], we applied text-mining techniques such as word occurrence and *TF-IDF* [27] to determine the words’ importance to each of the communities. To guide the quantitative step, we created word clouds^3^ for each community, where the word size reflected occurrence and color intensity word importance.

#### 2.3.3 Step 3: Narrative analysis

We analyzed the contents of the tweets using a coding scheme based on narrative analysis [28]. A narrative is a way of framing events in a manner that embodies a judgement on their nature [29]. Users on Twitter tend to frame media content in different ways. Two different users can tweet the same link, but their tweets may embody different judgements depending on the text accompanying the link. Similarly, the meaning of a tweet may change when other users respond to it. Furthermore, when users collectively engage in the creation and circulation of content around a specific narrative, they contribute to the materialization of a public narrative [28] – a process also known as narrative exchange [30].

The narrative analysis in this study employed the constant comparative procedure[31], in which the word clouds from step 2 were used to analyze the profile texts and tweets. So, if the words *‘autism’* and *‘MMR’* were found to be important in a specific community, we analyzed the tweets including these words written by members of this specific community. We also studied the conversational context (i.e. reply-chains) to understand how users use narratives to engage with each other. We identified the main narratives by coding the tweets relative to the 25 most occurring words in each community and grouped those codes into coherent groups.

#### 2.3.4 Step 4: Network analysis

As a final step, we examined communication flows between communities, counting the number of times that retweets, quotes, or replies occurred in each of them. We aggregated our data into a new network that we visualized to show flows of tweets, retweets, quotes, and replies between different communities. We zoomed in on these flows to identify which narratives flowed from one community to another, or which narratives clashed when two communities were interacting.

## 3 Results

When we started retrieving our data, Dutch news media reported on vaccination becoming mandatory in Italy^4^, followed by opinion pieces and readers’ letters on whether this should be the case in the Netherlands too. A month later, the Dutch Health Council released a report in which they argued that vaccinating all children against the Rota-virus would lead to the highest health gain^5^. This further sparked the public debate on vaccination, where newspapers published more opinion pieces and readers’ letters about the topic. A substantial part of the Twitter debate (still) seemed to revolve around a public appearance of a columnist in 2016, expressing her doubts about vaccination and referring to the negative information she found online.^6^

### 3.1 Communities

**Fig. 1:**
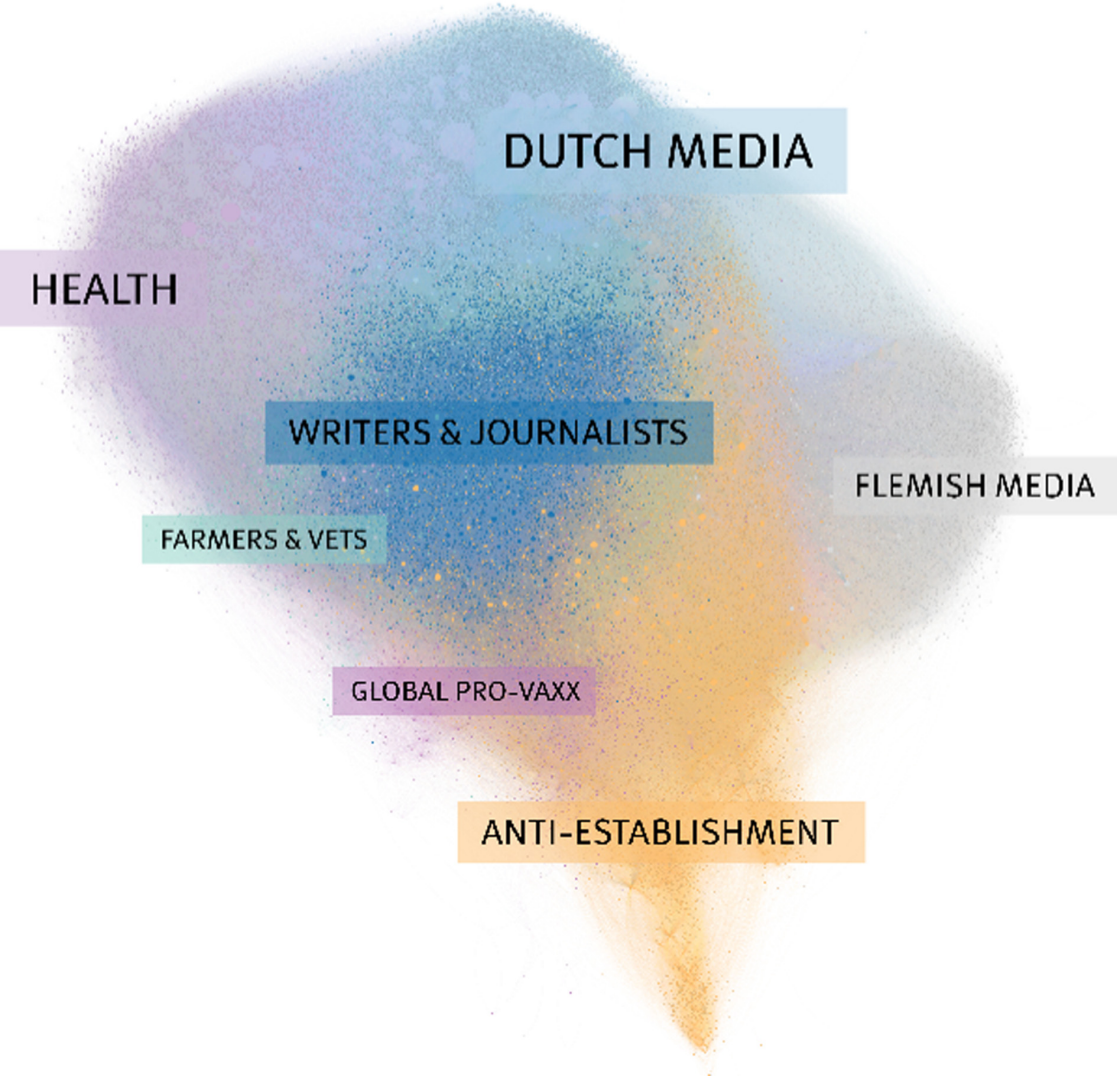
This network of Twitter users includes authors (users actively engaged with the vaccination debate) as well as their social context (users that the authors are generally following or are being followed by). The nodes (dots) represent Twitter accounts and are sized according to the number of incoming connections. They are positioned using Gephi’s ForceAtlas2 algorithm, which iteratively places nodes with many common connections close to each other. Connections between nodes are visualized using thin, transparent lines that collectively show larger paths between sections of the network. The community labels were added manually and were determined by analyzing the users’ profile texts during the text-mining step.

**Fig. 2:**
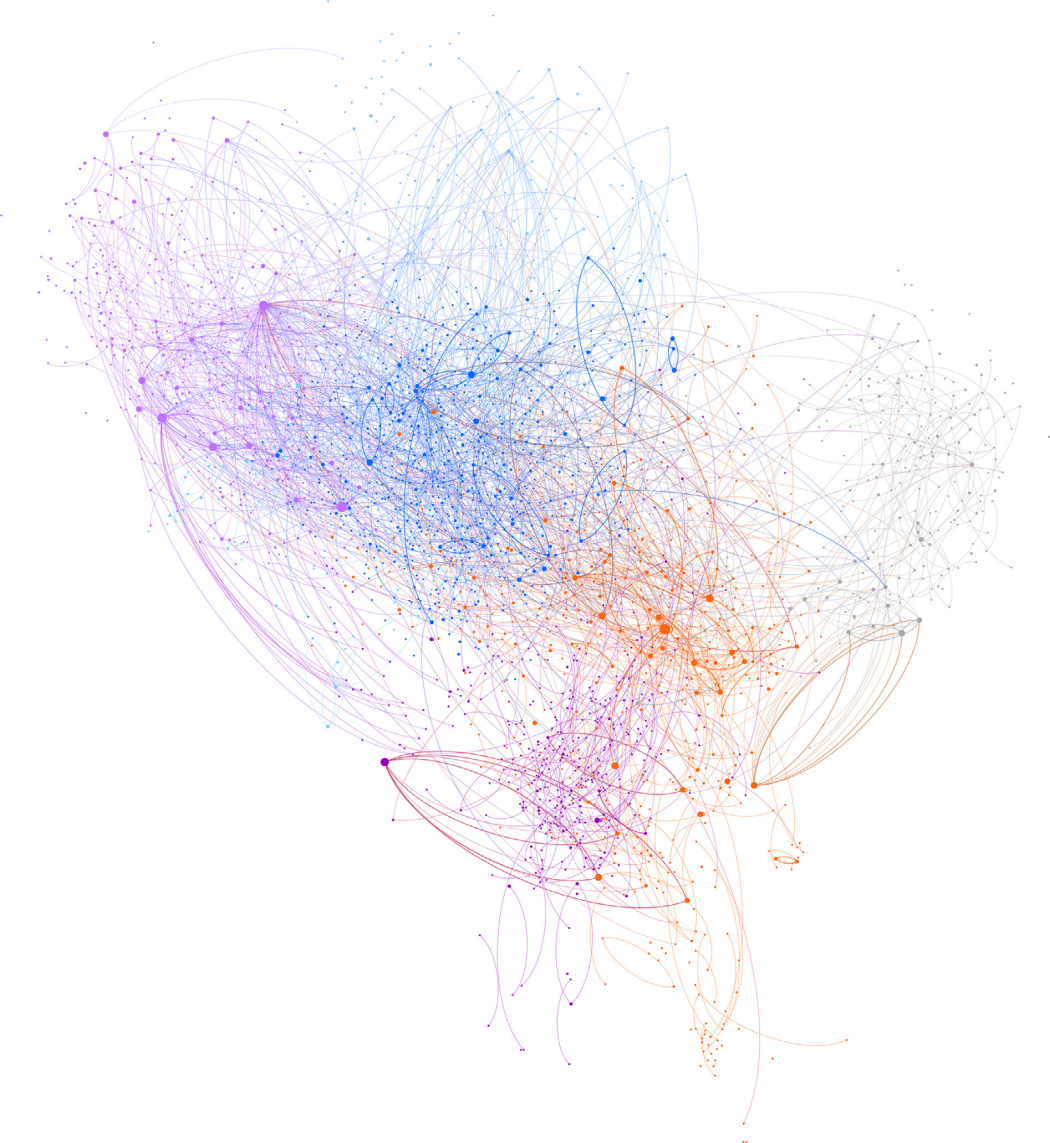
The network only including authors (users actively engaging with the vaccination debate). Edges represent interactions (retweets, quotes, mentions, and replies), node sizes represent the number of tweets about vaccination, and the colors corresponds with the communities.

The communities that were identified during the first step yielded a modularity statistic of 0.4, confirming that our network does indeed comprise multiple communities of densely connected users [32]. Fig. 1 contains a plot of the network in which the communities are distinguished by color. Note that Fig. 1 comprises authors as well as accounts that the authors generally follow or are being followed by. Fig. 2 only shows the authors in the network and how they interact with each other.

Table 1 includes properties of the different communities, comparing the number of authors with other users and distinguishing *initiators* (users that specifically mentioned vaccination) from *sources & responders* (authors of messages retrieved in the context of the debate). The engagement column shows the proportion of authors in each community.

**Table 1:** The characteristics of each community, specifying the proportion of authors, initiators (i.e.: users writing a tweet including one of the words from our search query), and sources & responders (i.e.: tweets that were retrieved in the context of the debate, such as the original messages retweeted by others and tweets that were replied to). The engagement column expresses the proportion of users from each community actively engaged in the vaccination debate. The percentages in the ‘totals’ row, express the proportions in the larger network.

	Total		Authors		Initiators		Sources & Responders		Engagement
	n	%	n	%	n	%	n	%	
Dutch Media	45,177	37.4%	154	6.2%	115	6.9%	39	4.7%	0.3%
Health	21,974	18.2%	437	17.5%	384	22%	53	6.4%	2%
Writers & Journalists	18,952	15.7%	689	27.5%	553	33.1%	136	16.4%	3.6%
Anti-Establishment	18,055	15%	512	20.5%	264	15.8%	248	30%	2.8%
Flemish Media	10,498	8.7%	230	9.2%	183	10.9%	47	5.7%	2.2%
Farmers & vets	3026	2.5%	115	4.6%	97	5.8%	18	2.2%	3.8%
Global Pro-Vaxx	2971	2.5%	363	14.5%	76	4.5%	287	34.6%	12.2%

Total	120,653		2,500		1672		828		2.1%

Table 2 shows the proportion of tweets, retweets & quotes, and replies about vaccination that the authors in each community produced.

**Table 2:** The characteristics of the tweets in our sample by community. The percentages in the ‘totals’ row, express the proportions in the larger network.

	Tweets		Retweets & Quotes			Replies			Avg. no. messages p. user
	n	%	n	%	% community	n	%	% community	
Dutch Media	233	4.5	16	1.6	6.9	22	1	9.4	1.5
Health	1,184	22.8	256	26.3	21.6	400	18.2	33.8	2.7
Writers & Journalists	1,252	24.1	198	20.3	15.8	661	30.1	52.8	1.8
Anti-Establishment	1,412	27.2	307	31.6	21.7	680	30.9	48.6	2.8
Flemish Media	438	8.4	64	6.6	14.6	209	9.5	47.7	1.9
Farmers & vets	177	3.4	31	3.2	17.5	50	2.3	28.3	1.5
Global Pro-Vaxx	500	9.6	101	10.4	20.2	175	8	35	1.4

Total	5,196		973		18.7%	2,197		42.3%	

To understand the nature of the communities, we analyzed the profile texts of all the users in each community. The descriptions below, reflect to the wider social contexts of the authors in each community.

#### 3.1.1 Dutch media (the Netherlands)

We identified a *Dutch media* community, comprising the country’s main news platforms, broadcasting organizations, and public personae such as columnists, presenters, politicians, and musicians. Within this community, these accounts are followed by entrepreneurs, freelancers, consultants, and public administration officials who are (professionally) interested in communication, politics, and media.

Although the *Dutch media* community is large – it spans 37.4% (n = 45,177) of the network – it is rather passive when it comes to tweeting about vaccination: 6.2% (n = 154) of all the authors reside in the *Dutch* media community, ultimately producing 4.5% of all the tweets (n = 233), 1.6% of all the retweets and quotes (n = 16), and 1% of all the replies (n = 22). Just 0.3% of the *Dutch media* community was engaged in the vaccination debate, writing an average of 1.5 messages per users, which are the lowest figures for all communities.

#### 3.1.2 Health

The *health* community was the second-largest community identified and is inhabited by general practitioners, nurses, consultants, and other health-care professionals working for hospitals, municipal health services, education, and mental health services. It also includes the official Twitter accounts of hospitals and public-health services.

The *health* community spans 18.2% (21,974) of the network and has an active core of 437 users, representing 17.5% of all the authors. The community wrote a relatively high proportion of tweets (n = 1184; 22.8%) and retweets and quotes (n = 256; 26.3%), but replied relatively little, (n = 400; 18.2%). 2% of the *health community* members engaged in the vaccination debate, which is the second lowest of the communities, writing an average number of 2.7 tweets, which is the second highest.

#### 3.1.3 Writers and journalists

The third largest community we identified is the *writers and journalists* community, which has a strong resemblance to its *Dutch media* counterpart. The community revolves around a group of (independent) journalists working for quality Dutch newspapers such as *‘de Volkskrant’* and *‘NRC’*. The accounts of the newspapers themselves, however, inhabit the *Dutch media* community. Generally, the journalists in this community do not actively tweet about vaccination, but are followed by individuals who do. These followers are a crowd of (professional) writers, communication professionals, entrepreneurs, education professionals, and public administration professionals who are interested in media and politics and sometimes engage in discussions on vaccination.

The *writers & journalists community* spans 15.7% (n = 18,952) of the network and has a large active core spanning 27.5% of all its authors (n = 689), producing a slightly smaller proportion (n = 1252; 24.1%) of all the Twitter statuses. The community retweets relatively little, producing 20.3% (n = 198) of the retweets and quotes, but produced a relatively large number of the replies (n = 661; 30.1%). With an average number of 1.8 tweets per author, the authors in *the Writers & Journalists* community produce a comparatively small number of tweets and of which a substantial part is replies, possibly indicating a conversational Twitter style and an incidental kind of involvement. 3.6% of the *Writers & Journalists community* engaged on the topic, which is the second highest figure of the communities.

#### 3.1.4 Anti-establishment

The fourth largest community we identified is the *anti-establishment* community, comprising homeopathy advocates, independent bloggers, alternative media, and users following these accounts. The followers generally do not disclose much about their real-world identity. The community’s members seem to come from the Netherlands, the United Kingdom, and the United States. In their profile descriptions, the community’s members: describe themselves as right-wing, patriotic, and conservative; promote a strong Dutch national identity; agitate against Islam; advocate for the Netherlands leaving the European Union (‘Nexit’); claim to have solidarity with Israel; praise Donald Trump; and state that they are looking for ‘the truth’ beyond the mainstream media. The users engaging on vaccination within this community seem to include conspiracy thinkers and homeopathy advocates, of whom the latter especially seems to form an odd minority in this community. Strikingly, the community also includes accounts with profile descriptions in Arabic or the Cyrillic script.

The *anti-establishment* community spans 15% (n = 18,055) of the network. The *anti-establishment* community is very involved: the active core spans 20.5% (n = 512) of all the authors, collectively producing the largest proportion of tweets (27.2%; 1412). The community produces comparatively large proportions of retweets and quotes (31.6%; n = 307) and replies (30.9%; n = 680). With 2.8% of the community engaging on vaccination, the *anti-establishment* community is the third-most engaged community. The *anti-establishment community* is the most vocal, writing an average number of 2.8 tweets per author.

#### 3.1.5 Flemish media (Belgium)

The fifth largest community we identified is similar to the *Dutch media* community, but concerns media accounts from Flanders in Belgium that are also in Dutch. The *Flemish media* community includes the accounts of broadcasting organizations, news platforms, NGOs, political parties, and universities, as well as the accounts of public personae such as politicians, artists, scientists, and athletes.

The *Flemish media* community is rather small, spanning 8.7% (n = 10,498) of the network and including 9.2% (n = 230) of all the authors, writing 8.4% (n = 438) of all the tweets. With 2.2% of the community members engaging on vaccination, writing an average number of 1.9 messages, the *Flemish media* community is the second-least engaged, but third-most vocal community.

#### 3.1.6 Farmers and veterinarians

The sixth largest community concerns a small cluster of farmers, veterinarians, and agricultural and horticultural organizations. The community spans 2.5% (n = 3,026) of the network, with an active core encompassing 4.6% (n = 115) of all the authors. This community produced 3.4% (n = 177) of all the tweets, 3.2% (n = 31) of the retweets and quotes, and 2.3% (n = 50) of the replies. 3.8% of the community actively engaged with the vaccination debate, writing an average number of 1.5 tweets. Although the community is the most engaged, they interact relatively little with others – probably because the *Farmers & Vets* community is talking about vaccination from a livestock perspective.

#### 3.1.7 Global media and vaccine advocates

Lastly, we identified a *global media and vaccine advocate* community containing: news platforms such as The Guardian, MSNBC, and Le Monde; health and development institutions such as the WHO, UNICEF, and the British Medical Journal; and public personae such as researchers, correspondents, artists, and athletes mainly based outside the Netherlands. In addition to these health organizations, the active core of the community includes independent bloggers, physicians, and pediatricians who can be regarded as pro-vaccination advocates, as they signify this with ‘#vaccineswork’ in their profile descriptions. Within this community, they are in turn followed by a minority of non-Dutch *anti-vaxxers* and conspiracy thinkers*,* whose messages were retweeted in the *anti-establishment* community. The *global media and vaccine advocate* community includes accounts tweeting about vaccination in English, Spanish, French, and Dutch.

The *global media and vaccine advocate* community spans a mere 2.5% (n = 2971) of the network, but includes 14.5% (n = 363) of all the authors. The community is very active, producing 9.6% (n = 500) of all the tweets, but merely 10.4% (n = 101) of all the retweets and quotes, and 8% (n = 175) of all the replies. The fact that 36.67% (n = 287) of the authors in the *global media and vaccine advocate community* were responsible for tweets retrieved in the context of the debate, possibly indicates that the *global media and vaccine advocate community* was mostly used as a source community for retweets and replies. With 12.2% of the community actively engaging on vaccination, the *global media and vaccine advocate* community shows the highest involvement with the topic of vaccination.

### 3.2 Narratives

After analyzing the different profile texts of all members of the online communities, we moved to the analysis of the vaccine-related tweets to determine the different narratives being circulated within and across communities.

#### 3.2.1 Scientific evidence

The *scientific evidence* narrative entails the circulation of news articles and peer-reviewed research papers that aim to show that vaccination works. The narrative is common in the *health* community, where members share peer-reviewed articles or refer to them in their replies to other users. The *scientific evidence* narrative is also common in the *Dutch media*, *Flemish media*, and *global media and vaccine advocate* communities, where news outlets and individual users share news about scientific studies or reply to statuses of (non-Dutch) anti-vaxxers. In turn, these tweets are often retweeted in the *health* community.

#### 3.2.2 Extremism

The *extremism* narrative implies that anti-vaxxers are extremists who have decided to ignore scientific information about vaccination. This narrative mostly concerns tweets, replies, and quotes, and often involves jokes, ridicule, and insults. One Twitter user, for example, compared individuals who do not vaccinate their children on religious grounds to religious fundamentalists, while another user compared being anti-vaxx to believing in well-known conspiracy theories such as the *Illuminati*^7^, *chemtrails*^8^, and *reptilian humanoids*^9^. The *extremism* narrative is especially dominant in the *writers and journalists* community, but also occurs in the *health* and *anti-establishment* communities. This shows that the latter community is not necessarily against vaccination – many users among the anti-establishment openly disagree with anti-vaxx messages.

#### 3.2.3 Information

The *information* narrative includes practical information about vaccination programs or announcements of informative events about the topic. Announcements are often posted directly by the Twitter accounts of health organizations in the *health* community, but also appear as news articles or press releases in the *Dutch media* and *Flemish media* communities. The statuses are often retweeted by members of the *health* community.

#### 3.2.4 Framing

The *framing* narrative is a form of critiquing the media, where Twitter users suggest that Dutch media should use different imagery to illustrate news items about vaccination. In response to a news article with a picture of a crying child, for example, one Twitter user replied: “*That picture is really a pity; it creates resentment of vaccination. It spreads a dangerous sentiment!*” The *framing* narrative is most common in the *writers and journalists* community, where it is retweeted frequently, but it also occurs in the *health* community.

#### 3.2.5 Natural medicine

The *natural medicine* narrative implies that vaccination is unnatural and is therefore harmful, unlike natural medicine or homeopathy. The narrative includes hyperlinks to external and often seemingly trustworthy websites about natural medicine or homeopathy, but also includes tweets that are characterized by twisted logic. One Twitter user, for example, asked rhetorically: “*Have you ever wondered why it is not allowed to use mercury in thermometers, but why it is allowed to inject it into babies?*” The narrative is dominant in the *anti-establishment* community and is often replied to fiercely by members of the *writers and journalists* community, who employ the *extremism* narrative.

#### 3.2.6 Survival of the fittest

The *survival of the fittest* narrative implies that vaccination weakens the human race, because it compromises the human gene pool with the offspring of people who are not naturally resistant to the diseases that people are vaccinated against. The narrative has a radical undertone of evolutionary extremism and exclusively occurs in the *anti-establishment* community.

#### 3.2.7 Freedom

The *freedom* narrative implies that compulsory vaccination is an infringement of personal integrity. Most of the narrative’s statuses surfaced in response to news about certain vaccinations becoming obligatory in Italy. Twitter users in the *anti-establishment* community reacted particularly angrily and called it an infringement of personal integrity, backing up their claims with links to national and European laws. In other cases, Twitter users compare compulsory vaccination to rape.

#### 3.2.8 Anti-religion

Traditionally, a strictly religious reformed minority in the Netherlands does not vaccinate their children for religious reasons. The *anti-religion* narrative implies that this is an excess of religious extremism. The narrative is common among the *writers and journalists* community as well as the *anti-establishment* community, meaning that the anti-establishment community is not necessarily *anti-vaxx*. Notably, however, it mostly surfaced as replies to debates about religion, meaning that vaccination was mentioned in response to tweets about religion, not as a conversation’s main topic.

#### 3.2.9 Conspiracy

The *conspiracy* narrative implies that vaccination is a conspiracy by the global elite to enable large pharmaceutical companies to maximize their profits. It includes quotes and links, often accompanied by short, rhetorical questions, such as: *“Isn’t it weird that the side effects of vaccines are only researched after the vaccines have been introduced already?”* An important role is played by dissenting voices such as parents, journalists, and scientists who speak out against vaccination. A good example is the online documentary *VAXXED,*^10^ to which members of the *anti-establishment* community often refer. The documentary contains interviews with (alleged) parents, journalists, and researchers and purports to show how governments and the pharmaceutical industry are trying to cover up overwhelming evidence against vaccination. The *conspiracy* narrative evokes replies from the *writers and journalists* and *health* communities, but counter-arguments from the *evidence* or *extremism* narratives are often dismissed as being the result of systematic brainwashing or an attempt to cover up the truth.

### 3.3 Interactions

Our analyses have thus far focused on separate communities, but we also wanted to determine how these communities interact with each other. We therefore aggregated the interactions between the communities to inspect these dynamics, as well as the circulation of community specific narratives through the network.

#### 3.3.1 Followers

The extent to which members of one community follow members of another determines the degree to which this community is exposed to the other. We have quantified how often community members follow members of other communities as a way to visualize information flows and examine patterns of exposure.

**Fig. 3:**
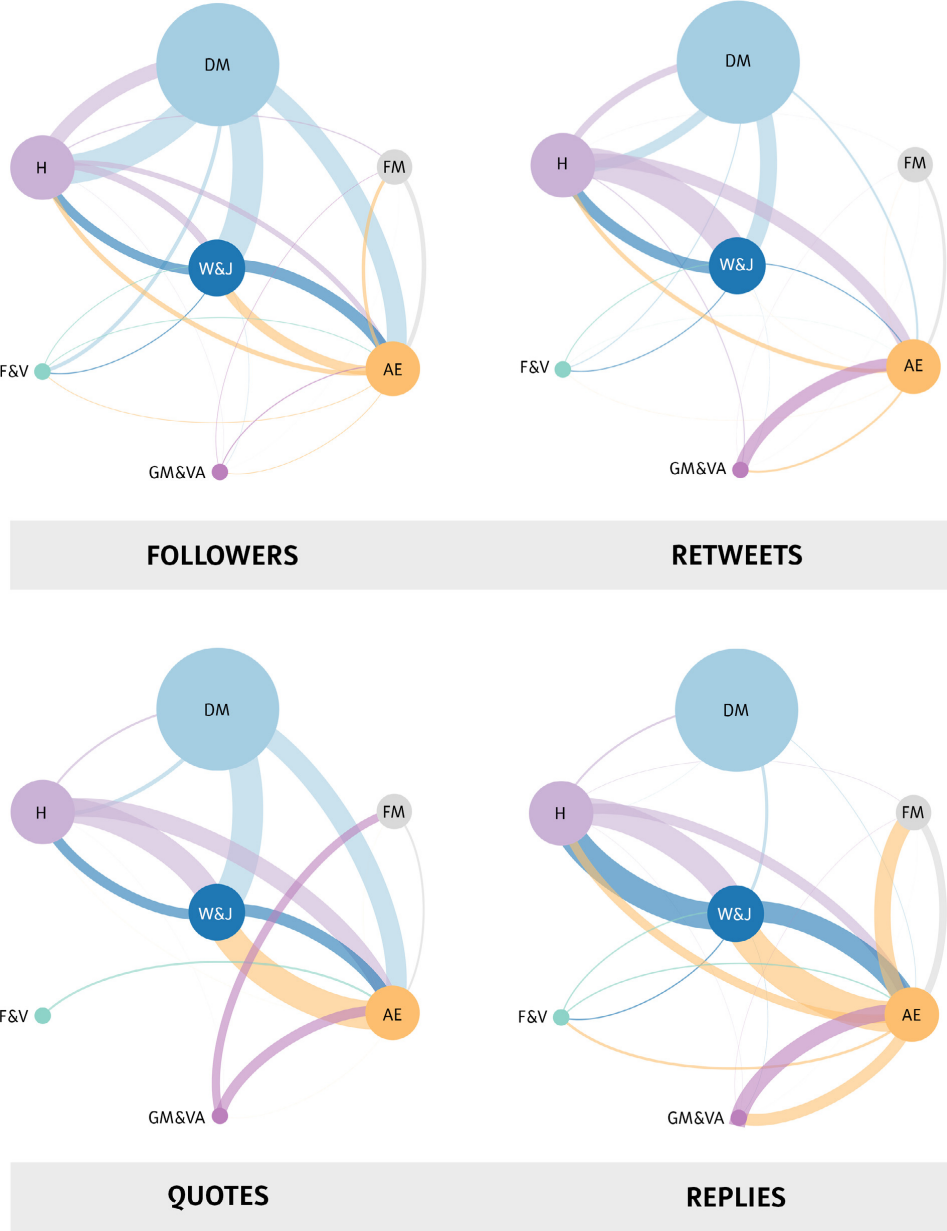
The connections between nodes represent interactions that enable content to flow from one community to another. Information flows clockwise, and connections are colored along to the community from which the content flows. For example, a light-blue line connecting the Dutch media (DM) community with the anti-establishment (AE) community means that: (1) the AE community is exposed to tweets from the DM community (followers); (2) the AE community is retweeting tweets from the DM community (retweets); (3) the AE community is quoting tweets from the DM community (quotes); and (4) the DM community is replying to the AE community (replies). (For interpretation of the references to color in this figure legend, the reader is referred to the web version of this article.) The connections between nodes represent interactions that enable content to flow from one community to another. Information flows clockwise, and connections are colored along to the community from which the content flows. For example, a light-blue line connecting the Dutch media (DM) community with the anti-establishment (AE) community means that: (1) the AE community is exposed to tweets from the DM community (followers); (2) the AE community is retweeting tweets from the DM community (retweets); (3) the AE community is quoting tweets from the DM community (quotes); and (4) the DM community is replying to the AE community (replies).

The top-left panel of Fig. 3 shows that the *Dutch media* community is widely followed by all the other communities, except the *Flemish media* and *global media and vaccine advocate* communities. The *Dutch media* community, however, rarely follows members of other communities back, except for members of the *health* community, where they follow a few public institutions. This characterizes the role of the *Dutch media* community in the vaccination debate as being an important information disseminator, mainly drawing information from traditional health institutions.

The *anti-establishment* and *health* communities follow each other, but are not as well-connected as with the *writers and journalists* community, which seems to play a central role in the vaccination debate.

Strikingly, the *anti-establishment* community is the only community that follows both the *global media and vaccine advocate* and *Flemish media* communities. This is a cosmopolitan trait that seems to be at odds with the profile of the *anti-establishment* community, but that mainly signify an interest in non-Dutch news organizations and interactions with the few *anti-vaccine activists* among the *global media and vaccine advocates* community.

#### 3.3.3 Quotes

Quotes are similar to retweets, but include the option to reframe the original message using a short piece of text. This makes it especially useful for retweeting statuses that users do *not* want to endorse. A community quoting another community may signify disagreement.

The *anti-establishment* community quoted the *Dutch media*, *health*, and *writers and journalists* communities, while only the *writers and journalists* and *global media and vaccine advocate* networks quoted them back. This means that the *health* and *Dutch media* communities did not respond when the *anti-establishment* community quoted (and criticized) their messages, while the *writers and journalists* and *global media* communities did. The *writers and journalists* community was actively engaged in conversation with the *health* and *anti-establishment* communities.

#### 3.3.4 Replies

Replies are similar to tweets, with the difference being that they would not have come into existence without the tweet they are responding to. So, replies signify interactions between people, but do not necessarily signify (dis)agreement.

Strikingly, the *Dutch media* community does not seem to be involved in the vaccination debate. The *writers and journalists* community communicated with the *health* and *anti-establishment* communities. The *anti-establishment* community also responded to the *writers and journalists*, *global media and vaccine advocate*, and *Flemish media* communities, and – to a lesser extent – the *health* community. The interactions between the *anti-establishment* community and the other communities are likely to signify fierce debate.

## 4 Discussion

In our study, we found 9 narratives of which 4 were negative about vaccination. By analyzing the wider social context of the authors of these tweets, we found seven distinct communities with unique profiles. In the debate on vaccination, authors in each community play a different role in circulating the narratives that we found: the *Dutch media* community focuses on news, but only happens to tweet about vaccination occasionally; the *health* community shares vaccination-related news, research, and other information; and the *writers and journalists* community criticizes the *Dutch media* community for representing vaccination in a negative way, and ridicules the *anti-establishment* community for conspiracy thinking. The *anti-establishment* community has mixed-feelings about vaccination: a minority of homeopathy advocates favors natural medicine over vaccination, while other members object vaccination for reasons that are often related to a distrust of traditional institutions and ‘the elite’, and others do not object at all and actually join the *writers & journalists* community in ridiculing their peers. Tweets from the *global media and vaccine advocate* community are mostly in favor of vaccination, but also include a few tweets from international anti-vaxxers. Both kinds of messages are retweeted, quoted or replied to in the *anti-establishment* community. The *Flemish media* and *farmers and vets* communities do not play a role of topical significance.

Examination of the interactions between the communities give the impression that the *health* and *Dutch media* communities employ a classic mass-media sender-approach, sharing news and evidence from a top-down perspective and hardly engaging in any one-to-one interaction. By contrast, the *global media and vaccine advocates* community is highly visible among the *anti-establishment* by actively responding to myths and misconceptions, and providing (retweetable) evidence to debunk these myths. The *writers and journalists* and *anti-establishment* actively interact with each other. The relationship between the *writers and journalists* and *anti-establishment* communities is troublesome, as the quotes and replies flowing between them are characterized by a great deal of ridicule and insults.

Our study shows that negative messages about vaccination are most prevalent in the *anti-establishment* community, but these messages are also often contested. The authors in the *anti-establishment* community include homeopathy advocates and conspiracy thinkers. They are often criticized or antagonized by their peers and by members of other communities using the *extremism* narrative, that seems to persist anti-vaxxers and conspiracy thinkers in their beliefs about the ‘arrogance of the elite’. This provides further empirical evidence for Kata’s argument that anti-vaxx communities are rooted in a post-modern worldview that distrusts traditional institutions [6] and favors personal narratives over scientific evidence [4]. Meanwhile, the fact-driven *research* narrative is mostly shared within the *health* community and does not reach the audience that apparently needs it the most.

Notably, in the *anti-establishment* and *global media and vaccine advocate* communities, we also found trails that might indicate troll activity (i.e. high-frequency tweeters, Cyrillic and Arabic scripts). Researchers have previously identified *‘content farms’* and *‘troll armies’* that purposely try to sow unrest in relation to themes that are controversial in the West. Apparently, vaccination has been targeted as one of these playing fields [33]. In future studies, therefore, researchers could use troll farm detection algorithms to distinguish communities of actual people from bots and trolls.

## 5 Conclusion

Our study provides insight into the main stakeholders in the Dutch vaccination debate on Twitter. We disentangled the different voices in the debate on vaccination by looking at shared elements in the wider media ecologies of users tweeting about vaccination.

Due to selective exposure, members of the *anti-establishment* community are more likely to be exposed to messages that are negative about vaccination than others. To effectively reach the group, Dutch health organizations could try to engage in an open dialogue to address questions, doubts, and worries; and by making information to debunk those myths more easily accessible and shareable. As such, our results provide helpful insight to developing public advocacy strategies in support of vaccination services [34].

The method we present in this study, and the scripts that we have made available publicly via GitHub^11^, can help health organizations to attune their strategies to the different communities and narratives in debates around health topics.

## 6 Statements

*Funding:* This work is supported by a grant from the Friends Lottery (MediaLab Project). The data collection is supported by a small grant from the RIVM for the study of Dutch conversations about vaccination on Twitter for the External Advisory Committee on Vaccination Willingness.

*Contributors:* RL wrote the first draft of the manuscript. All the authors reviewed and edited the manuscript and approved the final version.

*Authorship:* All the authors attest that they meet the ICMJE criteria for authorship.

*Conflicting interest:* There are no conflicts of interest.

*Ethical Approval/Guarantor:* The study complies with the professional, ethical standards of Dutch academia. ERB approval is not required for this kind of study.

## Appendix A Supplementary material

Supplementary data 1

Supplementary data 2: https://data.mendeley.com/datasets/fjvk93bc5m/1

## Footnotes

1 https://github.com/roel-sbcc/Networked-Twitter-Conversations.

2 Dutch (synonyms) for *‘vaccine’*, *‘vaccination’*, and different tenses of *‘to vaccinate’ and ‘to be vaccinated’.*

3 See Appendix A.

4 https://www.ad.nl/buitenland/ouders-in-italieneuml-krijgen-boete-als-ze-hun-kinderen-niet-inenten~a4d9675e/.

5 https://www.nu.nl/gezondheid/4939490/gezondheidsraad-adviseert-jonge-kinderen-vaccineren-rotavirus.html.

6 https://www.rtl.nl/video/9a6cef1d-610b-41f2-8aa6-7bd934a3cca6.

7 https://en.wikipedia.org/wiki/Illuminati#In_conspiracy_theories.

8 https://en.wikipedia.org/wiki/Chemtrail_conspiracy_theory.

9 https://en.wikipedia.org/wiki/Reptilians#David_Icke.

10 http://vaxxedthemovie.com/.

11 https://github.com/roel-sbcc/Networked-Twitter-Conversations.
