# Supplementary material for: Mapping the Dutch vaccination debate on Twitter: Identifying communities, narratives, and interactions
Source: Vaccine X. 2019 Mar 21;1:100019. doi: 10.1016/j.jvacx.2019.100019 (PMC6717092; doi:10.1016/j.jvacx.2019.100019)
Supplement: Supplementary data 1 [file mmc1.docx]

# Appendix 1: Word clouds

| **Dutch media** |  |
| --- | --- |
| Descriptions  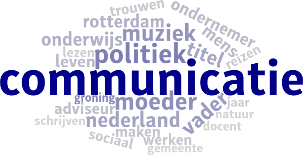 | Tweets  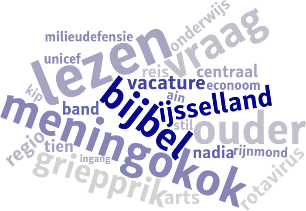 |
| **Health** | |
| Descriptions  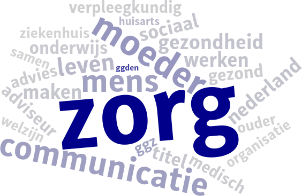 | Tweets  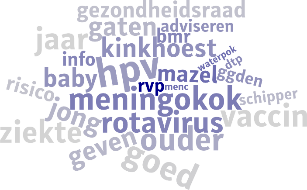 |

| **Writers and journalists** | |
| --- | --- |
| Descriptions  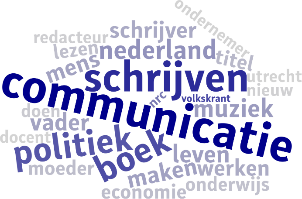 | Tweets  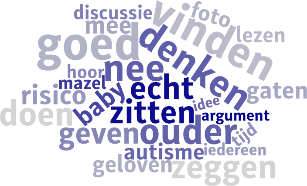 |
| **Anti-establishment** | |
| Descriptions  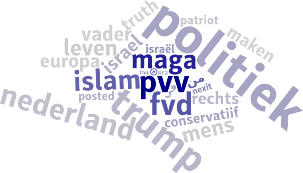 | Tweets  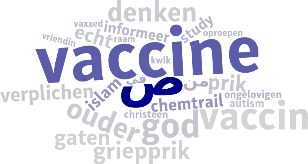 |

| **Flemish media** | |
| --- | --- |
| Descriptions  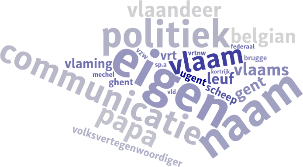 | Tweets  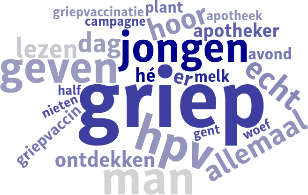 |
| **Farmers & vets** | |
| Descriptions  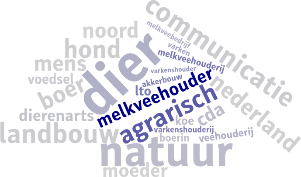 | Tweets  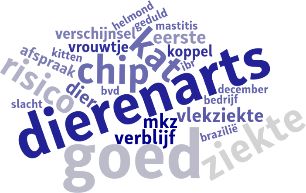 |

| **Global media and vaccine advocates** | |
| --- | --- |
| Descriptions  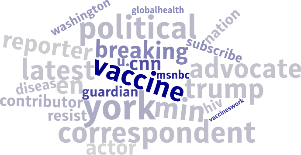 | Tweets  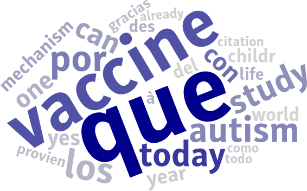 |
